# Vitamin D3 potentiates the antitumorigenic effects of arsenic trioxide in human leukemia (HL-60) cells

**DOI:** 10.1186/2162-3619-3-9

**Published:** 2014-03-25

**Authors:** Christian S Rogers, Clement G Yedjou, Dwayne J Sutton, Paul B Tchounwou

**Affiliations:** 1 Environmental Toxicology Research Laboratory, NIH-Center for Environmental Health College of Science, Engineering and Technology, Jackson State University, 1400 Lynch Street, Box 18540, Jackson, MS, 39217, USA

**Keywords:** Vitamin D3, Arsenic trioxide, Cytotoxicity, Apoptosis, HL-60 cells

## Abstract

**Background:**

Arsenic trioxide (ATO) is a novel form of therapy that has been found to aid acute promyelocytic leukemia (APL) patients. Our laboratory has demonstrated that ATO-induced cytotoxicity in human leukemia (HL-60) cells is mediated by oxidative stress. Pro-oxidants have been known to play a role in free radical-mediated oxidative stress. Vitamin D_3_, (Vit D_3_) an active metabolite of vitamin D has been reported to inhibit the growth of number neoplasms such as prostate, breast, colorectal, leukemia, and skin cancers. The goal of the present research was to use (HL-60) cells as an *in vitro* test model to evaluate whether low doses of Vit D_3_ potentiate the toxicity of ATO and whether this toxic action is mediated via apoptotic mechanisms.

**Method:**

HL-60 cells were treated either with a pharmacologic dose of ATO alone and with several low doses of Vit D_3_. Cell survival was determined by MTT assay. Cell apoptosis was measured both by flow cytometry assessment, and DNA laddering assay.

**Results:**

MTT assay indicated that Vit D_3_ co-treatment potentiates ATO toxicity in HL-60 cells in a dose dependent manner. A statistically significant and dose-dependent increase (p <0.05) was recorded in annexin V positive cells (apoptotic cells) with increasing doses of Vit D_3_ in ATO-treated cells. This finding was confirmed by the result of DNA laddering assay showing clear evidence of nucleosomal DNA fragmentation in vitamin and ATO co-treated cells.

**Conclusion:**

The present study indicates that Vit D_3_ potentiates the antitumor effects of ATO. This potentiation is mediated at least in part, through induction of phosphatidylserine externalization and nucleosomal DNA fragmentation. These findings highlight the potential impact of Vit D_3_ in promoting the pharmacological effect of ATO, suggesting a possible future role of Vit D_3_/ATO combination therapy in patients with acute promyelocytic leukemia (APL).

## Background

Acute promyelocytic leukemia (APL) is a subtype of the acute myeloid leukemia (AML). APL is characterized by abnormal, heavily granulated promyelocytes, a form of white blood cells. APL results in the accumulation of these atypical promyelocytes in the bone marrow and peripheral blood, and replaces normal blood cells. The standard treatment of this disease is chemotherapy and all trans-retinoic acid. Interestingly, arsenic trioxide (ATO) is a new form of therapy that has been found to benefit APL patients. Both *in vitro* and *in vivo* studies have shown that ATO can induce clinical remission of de novo and relapsed APL patients [1,2]. Several studies have reported that ATO induces apoptosis in malignant cells including APL, non-Hodgkin’s lymphoma, multiple myeloma, and chronic lymphocytic leukemia cells [3-5]. Also, ATO has been found to induce apoptosis in myeloid leukemia cells such as U937 and KG-1 cells [6]. ATO induced apoptosis is associated with the generation of reactive oxygen species that contribute significantly to cell killing [7-9] and inhibition of growth [10].

Vitamin D was discovered by Edward Mellanby in 1919 during his classic experiments with rickets [11]. It is classified into five forms including vitamin D_2_ (ergosterol); vitamin D_3_ (cholecalciferol); vitamin D_4_ (22, 23 dihydroergoalciferol); vitamin D_5_ (sitosterol [24-ethylcholecal- ciferol]); and vitamin D_6_ (stigmasterol) [12]. Vitamin D influences almost every cell in the body, and it is one of nature’s most potent cancer fighting agents. The receptors that respond to Vitamin D convert it to calcitrol which is a hormone. The Body organs use calcitol to repair damage and eradicate cancer cells. Experimental studies have shown that vitamin D is able to enter cancer cells and trigger apoptosis or cancer cell death. It is as effective at killing cancer cells in a way similar to the cancer drug Tamoxifen, and without the side effects.

A preclinical study indicated that exposing cancer cells and vascular endothelial cells to high concentrations of active metabolites of Vit D_3_ halts progression through growth arrest, apoptosis and cell cycle arrest *in vivo*[13]. Vit D_3_ potentiates the antitumor activity of a number of types of cytotoxic anticancer agents in *in vivo* preclinical models. Vit D_3_ analogues initiate signaling through a number of important pathways, but the pathway essential to the antitumor activities of Vit D_3_ are unclear [14]. Since both ATO and Vit D_3_ have been found to induce apoptosis in a variety of cancer cells, we designed this present study to evaluate the combined effect of both compounds. Also the mechanisms of action of VitD_3_ in combination with ATO for the treatment of APL remain largely unknown. Therefore, the aim of this research was to use human leukemia (HL-60) APL-cells as an *in vitro* test model to determine the potential mechanism of action of VitD_3_ on ATO chemotherapy of APL.

## Results

### Vitamin D3 potentiates the cytotoxicity of arsenic trioxide in HL-60 cells

**Figure 1:**
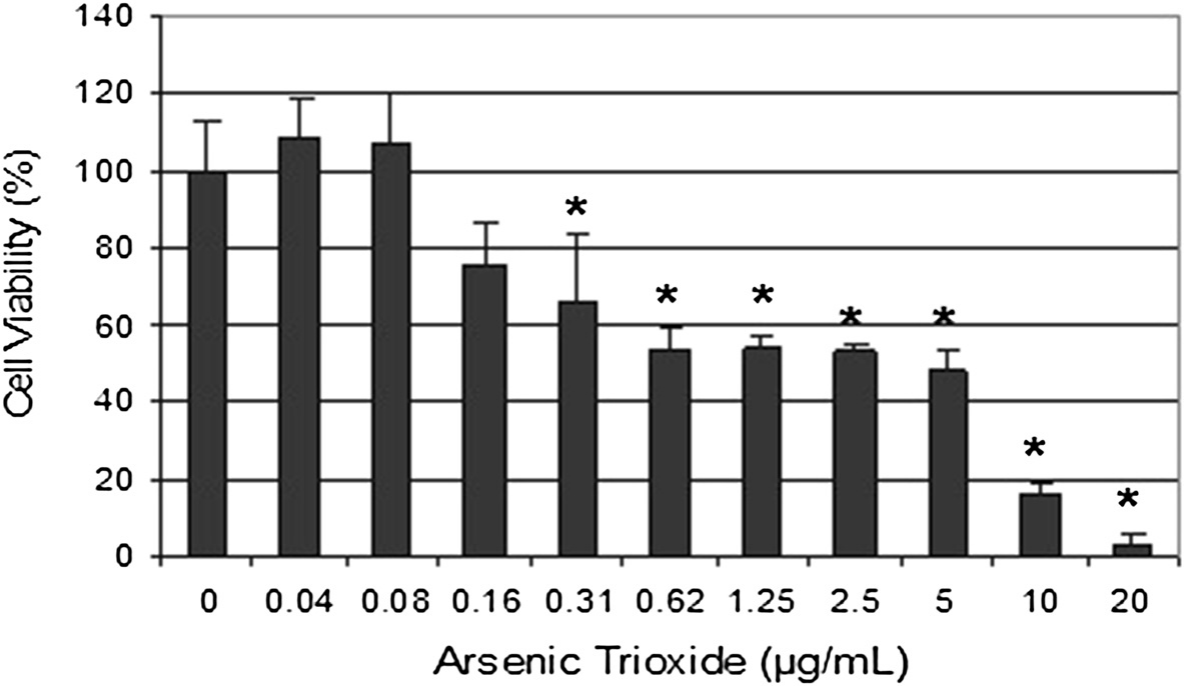
**Toxicity of arsenic trioxide to human leukemia (HL-60) cells.** HL-60 cells were cultured with different doses of arsenic trioxide for 24 hr as indicated in the Materials and Methods. Cell viability was determined based on the MTT assay. Each point represents a mean ± SD of 3 experiments with 6 replicates per dose. *Significantly different (*p <* 0.05) from the control, according to the Dunnett’s test [15].

**Figure 2:**
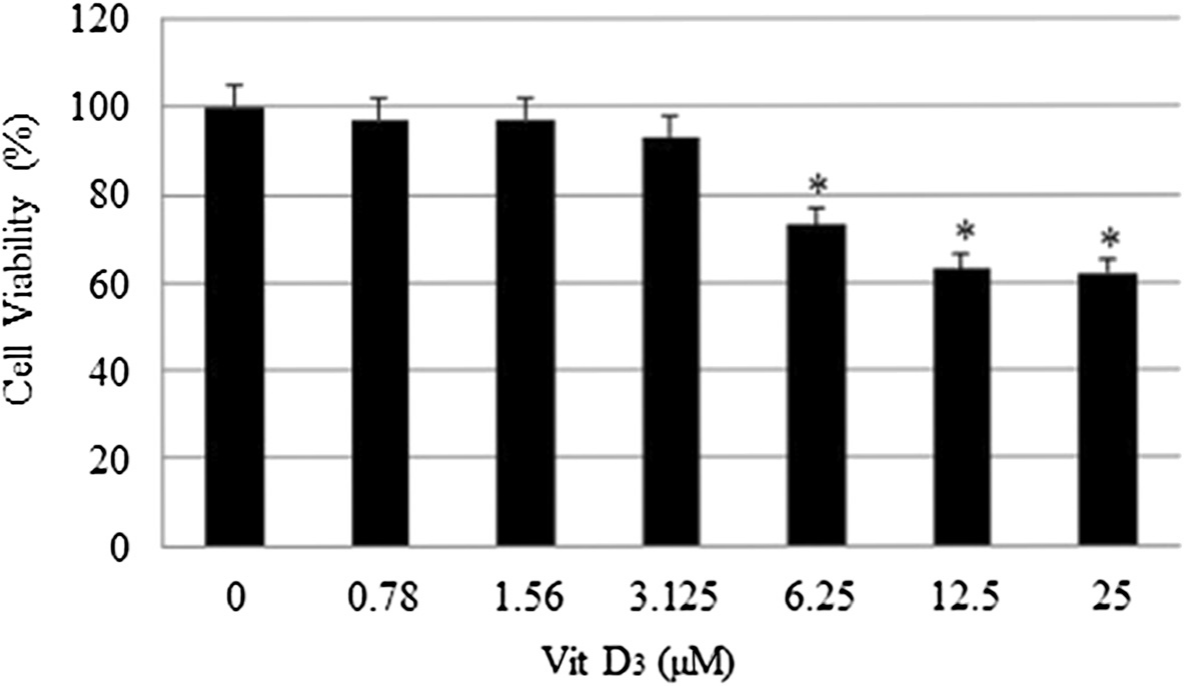
**Effect of vitamin D**_**3 **_**(Vit D**_**3**_**) on human leukemia (HL-60) cells.** HL-60 cells were cultured with different doses of Vit D_3_ for 24 hr as indicated in the Materials and Methods. Cell viability was determined based on the MTT assay. Each point represents a mean value and standard deviation of 3 experiments with 6 replicates per dose. *Significantly different (*p <* 0.05) from the control, according to the Dunnett’s test.

**Figure 3:**
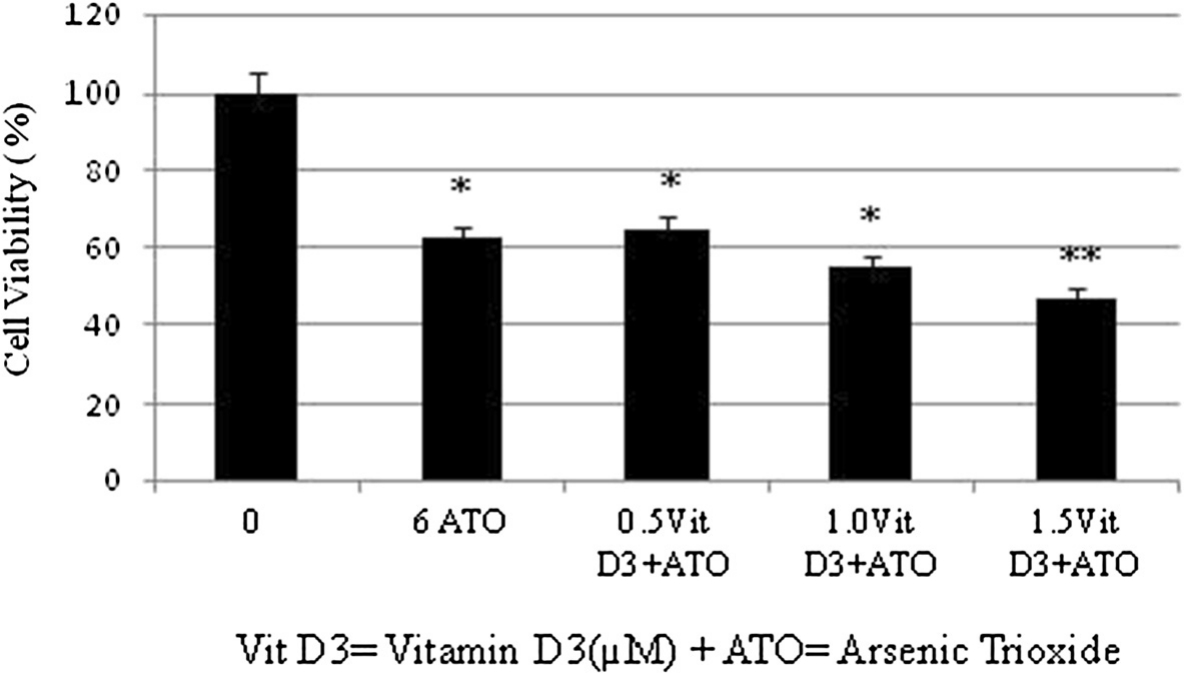
**Cytotoxic effect of VitD**_**3 **_**and ATO combination treatment on human leukemia (HL-60) cells.** HL-60 cells were cultured in the absence or presence of vitD3 and ATO or in combination of VitD_3_ and ATO for 24 hr as indicated in the Materials and Methods. Cell viability was determined based on the MTT assay. Each point represents a mean value and standard deviation of 3 experiments with 6 replicates per dose. *Significantly different from the control by ANOVA Dunnett’s test; *p* < 0.05. **Significantly different from ATO alone by ANOVA Dunnett’s test; *p* < 0.05.

We have previously reported that physiologic doses of ATO increase cellular proliferation while pharmacologic doses of ATO were highly cytotoxic to HL-60 cells, showing a 24 hr LD_50_ of 6.4 ± 0.6 μg/mL [15]. As shown in (Figure 1), a single pharmacologic dose (6 μg/mL) of ATO is highly cytotoxic to HL-60 cells. Low doses of Vit D_3_ have no effects on cell growth while on the other hand high doses inhibit the growth of HL-60 cells and cause significant cell death. Low doses of Vit D_3_ were selected based on the data generated from the MTT assay (Figure 2). Co-treatment of these cells using low doses (0.5-1.5 μM) of Vit D_3_ and a pharmacologic dose (6 μg/mL) of ATO resulted in a higher level of cell death than did ATO alone. We found that the viability of HL-60 cells declined from (62 ± 5)% in cells treated with ATO alone to (44 ± 3)% in cells co-treated with 1.5 μM Vit D_3_ and 6 μg/mL ATO with *P < 0.05* (Figure 3).

### Vitamin D3 potentiates arsenic trioxide-induced apoptosis in HL-60 cells

**Figure 4:**
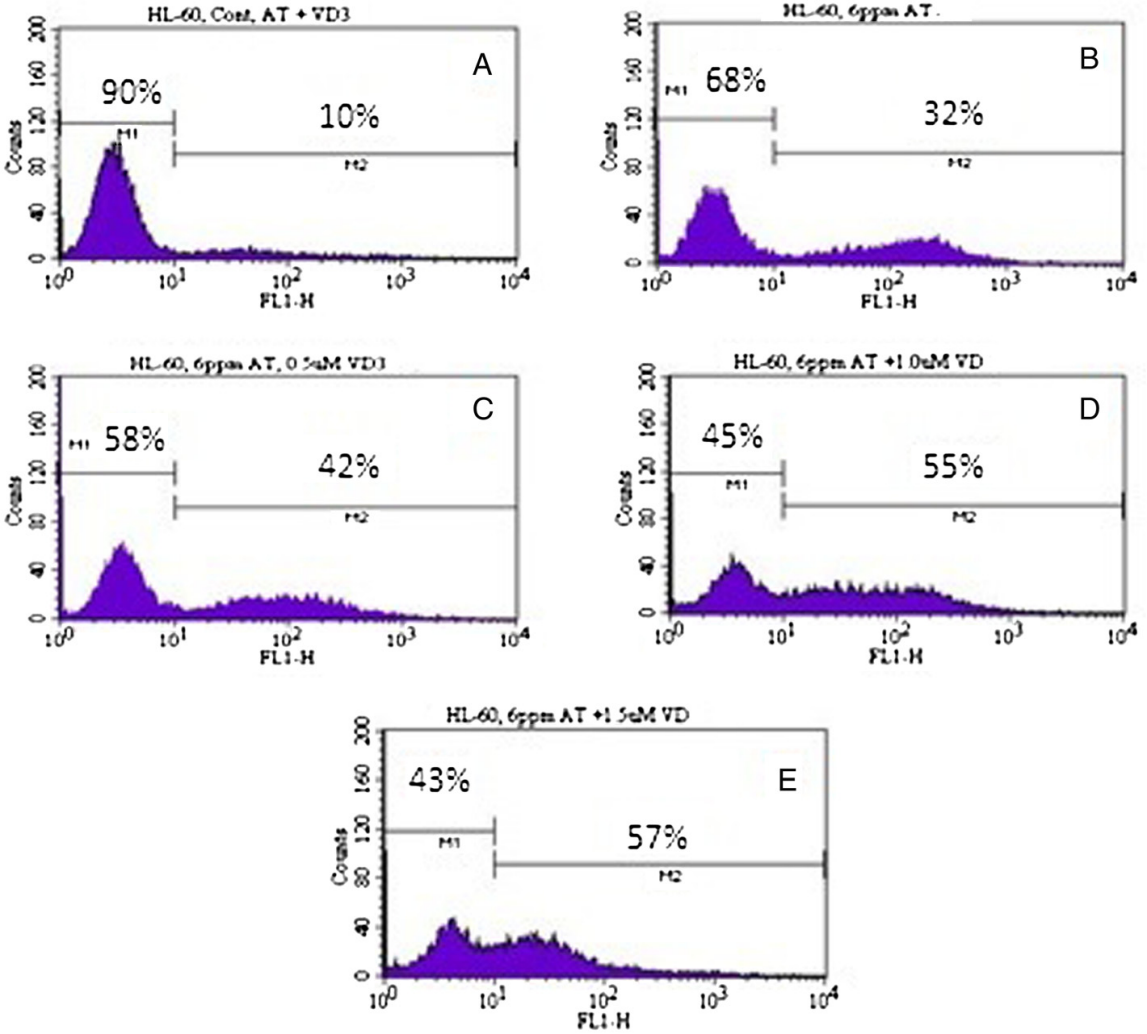
**Representative flow cytometry analysis data from Annexin V-FITC staining.** The histogram shows a comparison of the distribution of negative annexin V cells (M1) and positive annexin V cells (M2) after 24 hr incubation in HL-60 cells. A = control; B =6 μg/mL ATO; C = 0.5 μM VitD_3_ + 6 μg/mL ATO; D = 1.0 μM VitD_3_ + 6 μg/mL ATO; E = 1.5 μM VitD_3_ 6 μg/mL ATO.

**Figure 5:**
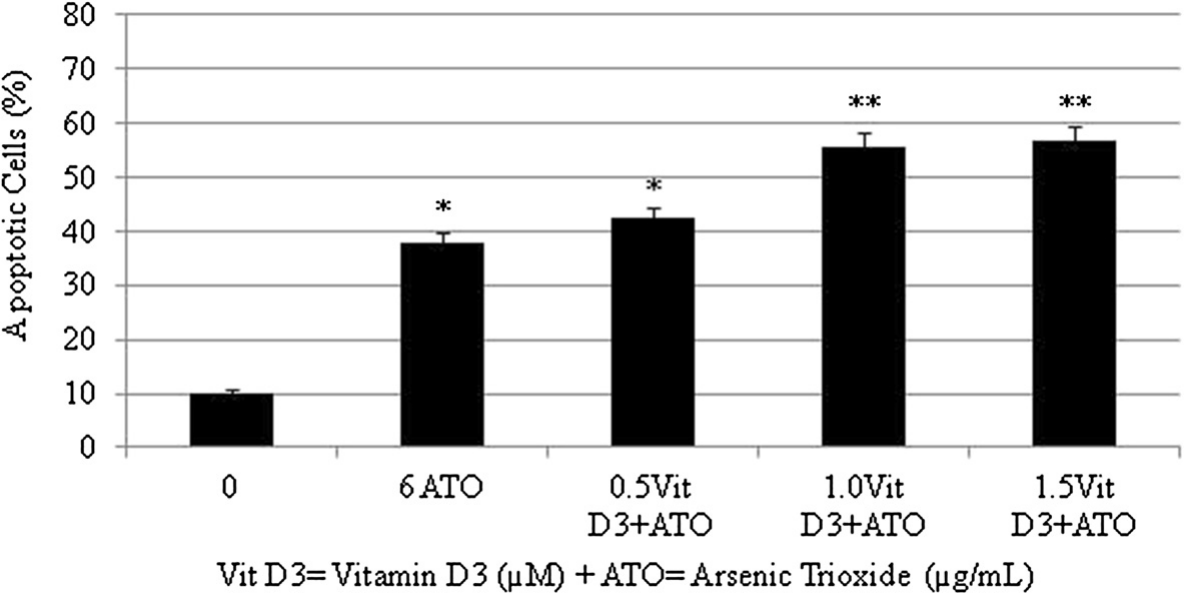
**Annexin V-FITC positive cells induced by either arsenic trioxide alone or vitamin D3 and arsenic trioxide combination in HL-60 cells.** Each point represents the mean value and the standard deviation of three experiments, showing similar results. *Significantly different from control (0 μg/mL), p < 0.05.

To determine whether low doses of VitD_3_ could sensitize arsenic trioxide (ATO)-mediated apoptosis, HL-60 cells were treated for 24 hr, subsequently stained with annexin V/PI, and analyzed by flow cytometry. As shown in (Figures 4 and 5), Vit D_3_ enhances the proportion of cells undergoing apoptosis in ATO-treated cells compared to ATO alone. For example, the proportion of annexin V positive was (38 ± 5)% in cells treated with 6 μg/mL ATO alone and (57 ± 6)% in cells treated with 1.5 μM Vit D_3_ plus 6 μg/mL ATO with P < 0.05.

### Vitamin D3 potentiates arsenic trioxide-induced nucleosomal DNA fragmentation in HL-60 cells

**Figure 6:**
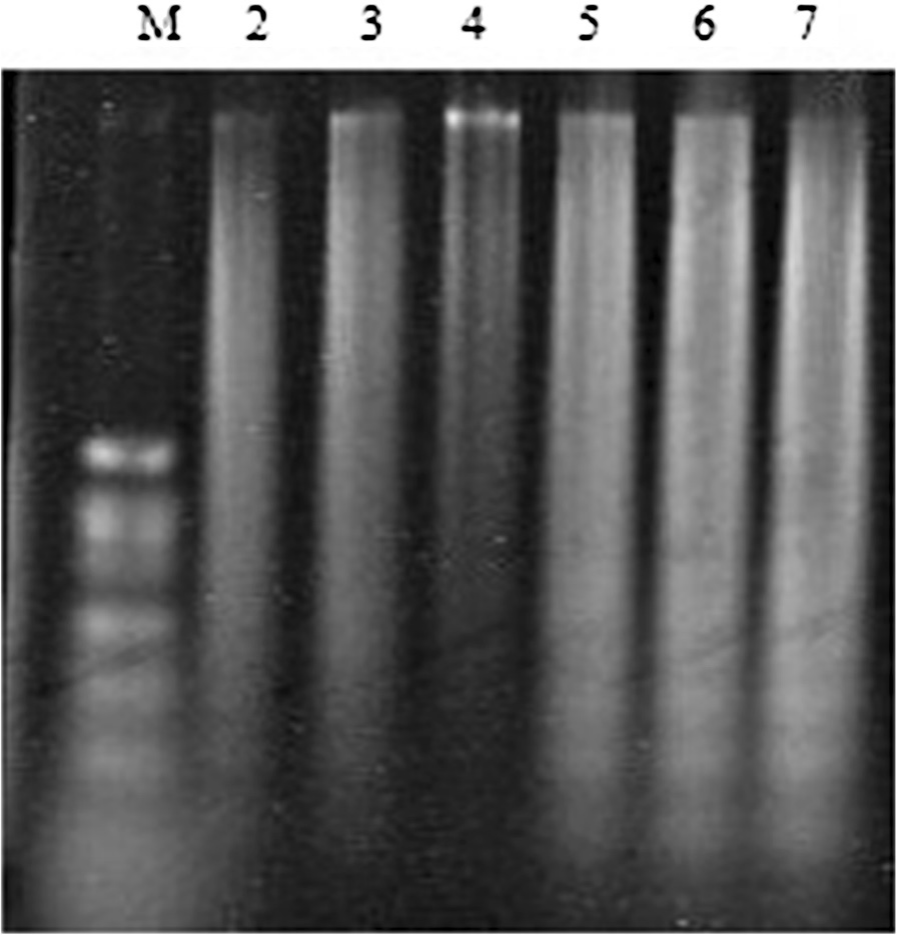
**Induction of DNA fragmentation in HL-60 cells.** Lane 1: M-Molecular weight marker. Lane 2-Positive control marker/Camptothecin (5 μM). Lane 3- ATO alone (6 μg/mL). Lane 4- Control with no treatment. Lane 5- VitD_3_ (0.5 μM) + ATO (6 μg/mL ATO); Lane 6- VitD_3_ (1.0 μM) + ATO (6 μg/mL ATO); Lane 7- VitD_3_ (1.5 μM) + ATO (6 μg/mL ATO). HL-60 celles were treated for 24 hr. The DNA gel was stained with SYBR Green 1 after electrophoresis on a 1% agarose gel and then analyzed on the Typhoon 9400 phospho imager.

In this study, we used camptothecin (CAM) as a positive control for DNA fragmentation because it is potent inhibitor of topoisomerase I and induces DNA fragmentation in a dose-dependent manner. Our results demonstrated positive DNA fragmentation with 6 μg/mL ATO alone and Vit D_3_ concentrations of (0.5, 1.0, 1.5 μM) and 6 μg/mL ATO combined, upon 24-hr incubation with HL-60 cells. As seen in the dose–response experiments of the present study, the extent of DNA fragmentation increased proportionately with increasing doses of Vit D_3_, suggesting that Vit D_3_ potentiates ATO induced apoptosis in HL-60 cells in a dose-dependent manner (Figure 6).

## Discussion

Several experimental studies have suggested a possible association between vitamin D and cancer risk reduction. For instance, *in vitro* and *in vivo* studies with cancer cells and tumors in mice found that vitamin D has several activities that might slow and/or prevent the development of cancer, including promoting cellular differentiation, decreasing cancer cell growth, stimulating cell death, and reducing tumor blood vessel formation [16-18].

Arsenic trioxide (ATO) has previously been reported to be cytotoxic to various mammalian cancer cell lines [10,19]. Data obtained from the present study indicate that the combination of low doses of vitamin D_3_ plus a pharmacologic dose of ATO is more cytotoxic to human promyelocytic leukemia (HL-60) cells compared to ATO alone. We found that Vit D_3_ co-treatment enhances ATO toxicity in HL-60 cells in a dose dependent manner. Consistent with the present study, previous by published reports from our laboratory demonstrated that ascorbic acid increases the activity of ATO in leukemia cells [20]. High doses of Vit D_3_ treatment alone prove to be cytotoxic, suggesting that it has the potential to be an enabling agent that sensitizes tumor cells to the cytotoxic effect of ATO-based chemotherapy. Vit D_3_ has been found to play an important role in controlling calcium homeostasis and bone metabolism [21,22] and in preventing diabetes [23]. Vit D_3_ induces monocytic differentiation in human myeloid leukemia cells including HL-60 myeloblastic cells [24].

To test the hypothesis that Vit D_3_ potentiation of ATO toxicity in cancer cells may be mediated via apoptosis, we assessed annexin V FITC/PI staining using the flow cytometry analysis. The flow cytometry data show a significant increase in annexin V positive cells (apoptotic cells) in Vit D_3_ co-treated cells with ATO compared to ATO alone (Figures 3 and 4). The percentage of annexin V cells positive was 38 ± 5% in cells treated with 6 μg/mL ATO and 57 ± 6% in those treated with 1.5 μM AA plus 6 μg/mL ATO with p < 0.05. By the means of flow cytometry, we report for the second time in our laboratory that antioxidant agents including ascorbic acid and Vit D_3_ potentiate the activity of ATO in cancer cells via phosphatidylserine externalization as result of the loss of membrane integrity, a major characteristic of cell death by apoptosis [10].

To confirm the apoptotic mechanism of Vit D_3_ on ATO, we further examined the apoptotic response, as judged by the appearance of a DNA ladder. A characteristic pattern of nucleosomal DNA fragmentation, which is the hallmark of apoptosis, was detected in ATO-treated cells alone and in Vit D_3_ plus ATO-treated cells (Figure 5). As seen in the dose–response experiment of the present study, the extent of DNA fragmentation increased proportionately with increasing doses of Vit D_3_, suggestive that Vit D_3_ potentiates ATO-induced apoptosis in HL-60 cells in a dose-dependent manner. Apoptosis usually is defined as programmed cell death and is characterized by specific morphologic features including cytoplasmic shrinkage, chromatin condensation, membrane blebbing, endonucleolytic degradation of genomic DNA, and the formation of apoptotic bodies [25,26]. There is evidence that apoptosis plays a role in the response of leukemia patients to chemotherapy, and there is probably an association between therapy-induced apoptosis and therapeutic efficacy [27,28].

Both the flow cytometry assessment and DNA laddering assay clearly demonstrated that co-administration of Vit D_3_ potentiated the apoptotic effects in ATO-treated HL-60 cells. Thus, confirming the enabling ability of Vit D_3_ on ATO. Although the mechanism by which Vit D_3_ enhances ATO-mediated cytotoxicity in HL-60 cells remains unknown, here we provided clear evidence that Vit D_3_ potentiates the antitumor effects of ATO, and this potentiation is mediated at least in part, via activation of phosphatidylserine externalization and nucleosomal DNA fragmentation.

## Conclusion

Given all the above, it is reasonable to infer that low doses of Vit D_3_ do not significantly affect cell viability *in vitro* while high doses of this compound are moderately toxic to HL-60 cells. We explore the cytotoxic and apoptotic effects of low doses of Vit D_3_ on the activity of ATO in human leukemia cells. The results of the MTT assay indicated that low doses of Vit D_3_ treatment significantly increase ATO toxicity in HL-60 cells in a dose dependent manner compared to ATO alone. This is most probably due to the different mechanisms through which both Vit D_3_ and ATO kill HL-60 cells. While testing the apoptotic mechanisms of these two compounds, both the flow cytometry assessment and DNA laddering evaluation showed an increase apoptotic cell death in cells co-treated with Vit D_3_ plus ATO compared to ATO alone, suggesting that Vit D_3_ acts as pro-oxidant in the presence of ATO. Given the recent therapeutic progress in the treatment of APL with ATO (Trisenox), we believe that the combination of Vit D_3_ plus ATO is a promising candidate for a novel therapeutic approach in the management of APL However further clinical trials are needed to confirm this finding.

## Methods

### Chemicals and test media

Arsenic trioxide, Lot No. 091419, CASRN 1327-53-3, MW 197.84, with an active ingredient of 100% (w/v) arsenic in 10% nitric acid was purchased from Fisher Scientific (Houston Texas). Vitamin D3, Lot No. 120M1635V was purchased from Sigma Aldrich (St. Louis, MO). Growth medium RPMI 1640 containing 1 mmol/L L-glutamine and fetal bovine serum (FBS) were purchased from Gibco BRL products (Grand Island, NY). Penicillin-Streptomycin, Lot No. 3000880, phosphate buffered saline (PBS-pH 7.4) Lot No. 3000892, and MTT assay kit Lot No. 3000800 were obtained from the American Type Culture Collection - ATCC (Manassas, VA). Annexin V-FITC Apoptosis Detection Kit Lot No. 42191 was purchased from BD Biosciences (San Diego, CA). Apoptotic DNA-Ladder kit, Lot No. 11324200 was purchased from Roche Molecular Biochemicals (Indianapolis, IN).

### Cell culture

The HL-60 promyelocytic leukemia cell line (No. CCL-240) was purchased from the American Type Culture Collection-ATCC (Manassas, VA). This cell line has been derived from peripheral blood cells of a 36-year old Caucasian female with acute promyelocytic leukemia (APL). The HL-60 cells grow as a suspension culture. In the laboratory, cells were stored in the liquid nitrogen until use. They were next thawed by gentle agitation of their containers (vials) for 2 minutes in a water bath at 37°C. After thawing, the content of each vial of cell was transferred to a 100 mm tissue culture dish, diluted with up to 20 ml of RPMI 1640 containing 1 mmol/L glutamine and supplemented with 10% (v/v) fetal bovine serum (FBS), and 1% (w/v) penicillin/streptomycin. The 100 mm culture dish, containing 2 × 10^6^ viable cells, were observed under the microscope, followed by incubation in a humidified 5% CO_2_ incubator at 37°C. Three times a week, they were diluted under same conditions to maintain a density of 5 × 10^5^/mL, and harvested in the exponential phase of growth.

### Treatment and measurement of cell viability

To explore the effect of vitamin (Vit D_3_) on the viability of HL-60 cells, 1 ml aliquots of cell suspension were transferred into 24 well tissue culture plates and treated with 1000 μM of Vit D_3_ diluted to reach final doses of .78, 1.56, 3.12, 6.25,12.5, and 25 μM cholecalciferol. Cells were placed in the humidified 5% CO_2_ incubator at 37°C for 24 hr. Cells incubated in culture medium alone served as a control (untreated wells). After incubation, 200 μL aliquots of MTT solution were added to each well and re-incubated for 30 min at 37°C, followed by low centrifugation at 800 rpm for 5 min. The supernatants were carefully aspirated and 200 μL aliquots of dimethylsulfoxide (DMSO) were added to each well to dissolve the formazan crystals, followed by incubation for 10 min to dissolve air bubbles. The culture plates were placed on a Multiskan micro-plate reader and the absorbance was measured at 570 nm. The amount of color produced is directly proportional to the number of viable cells. All assays were performed in three replicates for each, Vit D_3_, ATO, or Vit D_3_ + ATO concentration, and means ± SD values were calculated. Cell viability rate was calculated as the percentage of MTT absorption as follows: % survival = (mean experimental absorbance**/**mean control absorbance) × 100. In a recently published experiment, we reported that ATO is cytotoxic to HL-60 cells, showing a 24 hr LD_50_ of 6.4 ± 0.6 μg/mL [14]. Hence, to examine the effect of Vit D_3_ on ATO-induced cytotoxicity, cells were exposed to 0.5, 1.0 and 1.50 μM of VitD_3_ for 30 min and then co-exposed to 6 μg/mL ATO and incubated in humidified 5% CO_2_ incubator at 37°C for 24 hr, and tested for cell viability following the MTT assay protocol as described above.

### Flow cytometric analysis of phosphatidylserine externalization

The ability of cells to actively undergo apoptosis depends on their characteristic to lose membrane asymmetry in early phases of the apoptosis process. This early marker of programmed cell death is determined by analyzing phosphatidylserine (PS) translocation from the inner compartment of the plasma membrane to the outer compartment, thereby exposing PS to the external environment. Since Annexin V has a high affinity for PS it is effectively used in identifying apoptotic cells. Propidium iodide (PI) is a standard cytometric viability probe and is used to differentiate viable from nonviable cells. Viable cells with intact membranes exclude PI, whereas the membranes of dead and damaged cells are permeable to PI. HL-60 cells were double stained with a FITC-conjugated Annexin V antibody and PI (1 μg/mL) BD Biosciences (San Diego, CA) and analyzed by flow cytometry (FACSCalibur and Cell Quest Pro Software, BD Biosciences, San Diego, CA). HL-60 cells were treated with low doses of Vit D_3_ for 30 min prior and then treated with 6 μg/mL ATO for 24 hours. After treatment, 1 × 10^6^ cells/mL were washed with cold PBS and then resuspended in 1X binding buffer. One hundred (100) μL of the solution was transferred to a culture tube where 5 μL of both Annexin V-FITC and PI were added. The solution was mixed and then incubated for 15 min at room temperature in the dark. An additional 400 μL of 1X binding buffer was added to each tube and flow cytometric analysis was performed within 30 min of staining. A histogram analysis acquiring 10,000 events using M1 and M2 gates demonstrated viable cells versus nonviable cells. Results were analyzed and statistical analysis done using the Cell Quest Pro software BD Biosciences.

### Apoptosis DNA laddering assay

The purification of total DNA from HL-60 cells exposed to ATO alone and ATO + Vit D3 was done and DNA fragmentation analysis was performed to confirm the apoptotic mechanism as evidenced by DNA laddering, the hallmark of apoptotic cells. The apoptotic DNA Ladder kit from Roche Molecular Biochemicals (Indianapolis, IN) was used as directed. A sample volume of 800 μL containing 2 × 10^6^ cells was combined with binding buffer and incubated for 10 min at room temperature. Isopropanol was added to the sample and vortex. The sample mixture was added to the upper reservoir of the filter tube and centrifuged for 1 min at 8,000 rpm. The flow through was discarded and 500 μL of washing buffer was added to the upper reservoir and centrifuged for 1 minute at 8,000 rpm. The flow through was discarded and a final centrifugation was performed for 10 sec at 13,000 rpm to remove residual wash buffer. The filter tube was inserted into a clean 1.5 mL tube. The DNA was extracted by adding 800 μl of elution buffer and centrifuge for 1 min at 8,000 rpm. A higher dilution volume was used to increase the elution efficiency. Total DNA was measured by removing 1 μL from each sample and adding it to 999 μL of distilled H_2_O and reading the absorbance on a spectrophotometer. The optical density (OD) reading was multiplied by 50 μg/ml and the amount of DNA needed to add to the agarose gel was calculated to be 3 μL for each sample. The agarose gels were run in TBE (Tris-borate EDTA) buffer at 75 volts until the marker migrated to approximately 2 cm above the end of the chamber. The gel was stained with SYBR Green 1 for 24 hours and analyzed on the Typhoon 9400 phospho imager from GE Healthcare (Piscataway, NJ).

### Statistical analysis

Experiments were performed in triplicates. Data were presented as means ± SDs. Where appropriate, one-way ANOVA or student paired *t*-test was performed using SAS Software available in the Biostatistics Core Laboratory at Jackson State University. *P*-values less than 0.05 were considered statistically significant.

## Abbreviations

ANOVA: One way analysis of variance; APL: Acute promyelocytic leukemia; ATO: Arsenic rioxide; DMSO: Dimethylsulfoxide; DNA: Deoxyribonucleic acid; FACS: Fluorescence activated cell sorting system; MTT: 3-(4,5-dimethyl-2-thiazolyl)-2,5-diphenyl-2H-tetrazolium bromide; PS: Phosphatidylserine; Vit D3: Vitamin D_3_ .

## Competing interests

The authors declare that they have no competing interests.

## Authors’ contributions

CR and CY have performed the experiment and drafted the manuscript that was reviewed by all authors. CY and DS have assisted in performing the statistical analysis and data interpretation. P T has supervised the experiment and reviewed the manuscript for submission. All authors have read and approved the final draft of the manuscript.
